# Causal Rasch models

**DOI:** 10.3389/fpsyg.2013.00536

**Published:** 2013-08-23

**Authors:** A. Jackson Stenner, William P. Fisher, Mark H. Stone, Donald S. Burdick

**Affiliations:** ^1^MetaMetrics Inc.Durham, NC, USA; ^2^School of Education, University of North CarolinaChapel Hill, NC, USA; ^3^Graduate School of Education, University of CaliforniaBerkeley, CA, USA; ^4^Professor Emeritus, Department of Psychology, Aurora UniversityAurora, IL, USA; ^5^Distinguished Research Scientist, MetaMetricsDurham, NC, USA

**Keywords:** causality, models, prediction, assessment, reading ability, Rasch models, quantification, construct validity

## Abstract

Rasch's unidimensional models for measurement show how to connect object measures (e.g., reader abilities), measurement mechanisms (e.g., machine-generated cloze reading items), and observational outcomes (e.g., counts correct on reading instruments). Substantive theory shows what interventions or manipulations to the measurement mechanism can be traded off against a change to the object measure to hold the observed outcome constant. A Rasch model integrated with a substantive theory dictates the form and substance of permissible interventions. Rasch analysis, absent construct theory and an associated specification equation, is a black box in which understanding may be more illusory than not. Finally, the quantitative hypothesis can be tested by comparing theory-based trade-off relations with observed trade-off relations. Only quantitative variables (as measured) support such trade-offs. Note that to test the quantitative hypothesis requires more than manipulation of the algebraic equivalencies in the Rasch model or descriptively fitting data to the model. A causal Rasch model involves experimental intervention/manipulation on either reader ability or text complexity or a conjoint intervention on both simultaneously to yield a successful prediction of the resultant observed outcome (count correct). We conjecture that when this type of manipulation is introduced for individual reader text encounters and model predictions are consistent with observations, the quantitative hypothesis is sustained.

The thermometer, as it is at present construed, cannot be applied to point out the exact proportion of heat …. It is indeed generally thought that equal divisions of its scale represent equal tensions of caloric; but this opinion is not founded on any well decided fact.

Joseph-Louis Gay-Lussac (1802)

Thirty years ago, three of the authors of this article introduced the concept of the specification equation as a new model for measurement validity studies in the human and social sciences (Stenner and Smith, 1982; Stenner et al., 1983; Stone and Wright, 1983). The primary characteristic setting the concept of the specification equation apart from other approaches to psychological and social measurement is its decidedly mechanismic (causal) approach. In the years that have passed, the causal notions associated with the specification equations have been variously endorsed (Hobart et al., 2007) and ignored (Messick, 1989).

Perhaps the causal perspective on measurement would not be deserving of an update were it not for the theoretical fruitfulness and widespread adoption of the Lexile Framework for Reading. In this paper, the ideas presented in the initial papers are situated in the contemporary psychometric context and some recent advances are outlined. The framework for measurement in the human and social sciences set out here adopts from 17th- and 18th-century physical science measurement a focus on experimental manipulations of three variable systems (such as *F* = *MA*). We conjecture that in three-variable systems of this kind, demonstrating that all three variables are quantitative requires manipulations of one variable to result in predictable changes to a second variable when the third is held constant. When this manipulation works up and down the scale no matter which variable is held constant, then all three variables are quantitative.

For instance, given that a change in mass can be offset by a change in acceleration such that force is left unchanged, and this trade-off operates the same way at every point along the mass scale and along the acceleration scale, then mass, acceleration, and force are quantitative attributes. In what follows, we make some strong claims regarding the possibility that human and social science measurement might find foundations in causal relationships akin to those obtained in the physical sciences. Measurement is conceived in this context as a three-variable equation involving an attribute measure, a measurement mechanism and a measurement outcome (often a count). The primary conjecture is that a prescribed change in a mechanism (such as text complexity) is offset by a change in an attribute measure (such as reading ability) to hold a measurement outcome (percent correct) constant. Our unproven conjecture is that, when this trade-off functions precisely the same way up and down the scale, then the three variables are quantitative (equal interval) variables. We follow Pearl (2000, p. 158) in taking “the stand that the value of structural equations lies not in summarizing distribution functions but in encoding causal information for predicting the effects of policies.”

In other words, our purpose is to elaborate the intuition that if like differences in differences have the same predictive outcomes wherever along the scale the differences are experimentally induced, then the attributes are quantitative. A 100 L difference between text complexity and reader ability has the same implication for predicted success rates on reading items wherever along the scale the 100 L difference is experimentally induced. If the predicted success rates are observed, then 100 L means the same thing up and down the scale in the substantive terms of real texts encountered by real readers. Is not this capacity to infer the repetition of a qualitatively meaningful constant amount precisely what we mean by “equal interval”?

## Quantity vs. heterogeneous orders

Michell (1997, 2000, 2007) effectively made the case for what measurement is and in his earlier writings, exhorted psychologists to adopt this “standard model” in their practice. Over time, this eminently sensible call morphed into a diagnosis of so-called pathological behavior on the part of the field of psychology and its high priests and priestesses, called *psychometricians*. Most recently, some researchers have asserted that no psychological attributes have been shown to be quantitative and have offered the explanation that none will be because these attributes are actually heterogeneous orders:

Scientists who care more about appearing to be quantitative and the advantages that might accrue from that appearance, than they do about investigating fundamental scientific issues, put expedience before the truth. In this, they do not conform to the values of science and elevate non-scientific interests over those values, thereby threatening to bring science as a whole into disrepute. If the attributes that psychometricians aspire to measure are heterogeneous orders then psychometrics, as it exists at present, is fatally flawed and destined to join astrology, alchemy and phrenology in the dustbin of science. (Michell, 2012, 16)

Throughout this paper, we draw analogies to human temperature measurement via the NexTemp thermometer^1^ and to English reading ability measurement^2^ via the EdSphere™ technology (Hanlon et al., 2012). Both measurement systems share a basic three-part structure in common with many other measurement technologies (Hebra, 2010): (1) An observational outcome, often a count (number of NexTemp cavities changing from green to black reflecting temperature change or count of correct responses to EdSphere™ four-choice embedded cloze items reflecting change in reading ability), (2) a causal mechanism that transmits variation in the intended attribute (temperature or reading ability) to the observed outcome, and (3) an attribute measure denominated in some unit (such as degrees Celsius or Lexiles). As Michell (2012) correctly observed, “Only quantitative attributes can be measured because only they possess the necessary kind of homogeneity” (7). The crucial question is: How does one test for the necessary homogeneity that distinguishes quantity from mere order?

The history of science reveals a developmental course traveled by every attribute that figures in advanced scientific discourse. For some of these attributes, the full course took centuries and for others many decades, but in no case was an attribute born quantitative. Most quantities that are now recognized began as qualitative distinctions (hot, cold, or good reader, poor reader), were later understood to be ordinal and grew to adulthood as a quantity that admits of homogeneous differences. Historical and philosophical treatments that ignore this developmental pathway and the struggles along the way, whether purposeful or not, only confuse (Chang, 2004; Sherry, 2011). In ancient times, only extensive attributes (time, volume, weight, etc.) were considered measurable. Attributes such as temperature, density, and electromagnetism were in their infancy and were specifically not thought to be measurable (Heilbron, 1993; Roche, 1998).

It took hundreds of years of science and a solution to the particularly knotty problem of forsaking human sense impression as the final arbiter of disputes concerning quantitative attributes. In the case of temperature, degrees were obviously not homogeneous because *one more* had such ridiculously discontinuous consequences (e.g., a nominal one-degree change makes water freeze or boil). The lesson here is that perceived qualitative distinctions are a poor guide as to whether an attribute is quantitative as measured. Magnitudes of the same quantitative attribute appear to differ in dozens of ways. In the infancy and adolescence of many attributes that today are considered quantitative, *quantitative homogeneity* was impure. Quantitative measurement has been a hard-fought and dearly won battle; it has never been and never will be a given or something to be decided solely by speculation about perceived qualitative distinctions and heterogeneous orders. The historical record includes many attributes thought at one time to indicate mere order and later rendered as quantities and is void of confirmed quantitative attributes that are later found to be merely ordinal. This does not mean that every ordered attribute will, given time, be rendered quantitative, but science has taught us to leave the door ajar because, over and over, science has found ways to render some of today's *orders* as tomorrow's *quantities*.

Yet another argument is due to Sherry (2011): if treating an attribute such as text complexity or reading ability as quantitative allows one to employ mathematical models to bring order to the data, “then a plausible explanation for this success is that the attribute is approximately quantitative” (523).

So, if speculation about a hierarchy of states, stages, discontinuities, heterogeneous orders, and conceptual inclusions are unworthy guides to whether or not an attribute is quantitative, how does one proceed? As we will show, there is a simple, powerful test for essential homogeneity: the trade-off property. In many three-variable systems, such as, for example, a causal Rasch model that relates observed outcome (dependent variable) to a difference (or product) between the measurement mechanism (e.g., item difficulties) and the measurement attribute (e.g., reading ability), the trade-off property asserts that a manipulation on the measurement mechanism (e.g., increasing text complexity by 100 L) can be traded off for an offsetting manipulation on the measurement attribute (increasing reader ability by 100 L) to hold constant the observed outcome (percent correct).

We conjecture that when this trade-off property can be experimentally verified up and down the scale, all variables in the system are quantitative and differences (units) are monotonic, invariant, and homogeneous. As suggested by Trendler (pers. commun., 2011), and expanding on Burdick et al. (2006), the capacity to successfully predict differences from differences up and down a scale are an acid test for quantity. This trade-off property (in the service of successful substantive theory) is all that matters in demonstrating causality, deep understanding of the construct, and predictive control over interventions. There will always be extraneous qualities that can be identified in rhetorical arguments against a quantitative claim. Endless speculations can be made about why this or that feature of an attribute is a qualitative distinction worthy of notice (such as variation in the freezing point of water, relative to temperature), thus, appearing to render the unit non-homogeneous.

But such speculations are unable to offer distinctions that make a difference when evidence supports the trade-off property. This does not, however, mean that data fit to a descriptive (not explicitly causal) Rasch model implies that all three variables in the system are quantitative. Such fit may be suggestive in the same way that highly correlated data are a good place to look for causal relationships, but it is inconclusive as to the quantitative claim until experimental manipulation sustains the trade-off relation.

Perhaps an intuitively accessible illustration of the power of the trade-off relation would be helpful. Suppose Lexile reader measures and Lexile text complexity measures were replaced with randomly spaced numbers that preserve mere order. In this case, a text complexity difference between two articles might be increased from +10 L to 150 L and the adjacent 10 L article difference might be increased to 160 L. Thus, order is preserved but essential homogeneity is destroyed. Clearly, the trade-off relation would be violated even though strict order was maintained. Each trade off would produce wildly varying predicted observed outcomes, not the constant observed outcome asserted under the trade-off property. Order is not sufficient; differences must be preserved and retained as invariant ranges up and down the scale.

Note that there is a crucial difference between the trade-off property and order-restricted inference tests such as double and triple cancelation (Michell, 1997; Kyngdon, 2008). The trade-off relation is a claim about what will happen in the individual case or a token test of a causal model. Most tests of double and triple (and so on) cancelation with which we are familiar test between-person orders, not within-person (intra-individual) differences (but see von Winterfeldt et al., 1997; Luce, 1998). The trade-off property can and should be tested within persons over time. We further develop this idea later in the paper.

*Process talk* localizes the active features of a measurement process within the person or object of measurement. *Mechanism talk* conceptualizes the active ingredients as a tunable mechanism within the instrument. In temperature measurement, it seems awkward to talk about a response process that functions within the person and that interacts with the instrument to produce the cavity count on a NexTemp thermometer^1^. It is often more useful to reflect on the instrument features (tunable mechanisms) that transmit variation in the attribute to the cavity count. When the attribute *reader ability* is measured with a machine-generated, multiple choice, cloze test, the tunable mechanism involves the text complexity of the passage and the decision as to which words are “clozed” and how the foils are chosen. A causal Rasch model may be seen as formalizing how a measurement mechanism and an attribute measure cooperate to produce (cause) the observed outcome. The difference between calibrated mechanism and measured attribute causes the observed outcome. When viewed this way, it is clear that manipulations of the mechanism (e.g., added text complexity) can be offset (traded off) by a manipulation of the attribute (more practice reading) to hold the observed outcome (success rate or comprehension) and the measure based on it constant.

In this formulation of the measurement process, we require that the way the mechanism works and the way it trades off against the attribute measure to cause changes in the observed outcome must be invariant both within and between objects of measurement (e.g., persons). Note that if the mechanism is well explicated and functions invariantly across objects of measurement, it does not matter how the object arrived at its position on the attribute scale. Process talk can confuse on this point and may cause worry about the how. For example, two persons, Jane and Dave, may both have a fever of 104°F. For purposes of human temperature measurement (as opposed to, say, treatment regime), it is not relevant that Jane has a bacterial infection and Dave a viral infection. What is relevant is that temperature and the measurement mechanism cooperate in the same way for Jane and Dave, and for every other person, independent of what might have caused a deviation from normal human temperature. Similarly, two fourth-grade readers may both read at 800 L, but one got there with a particularly fortuitous genetic makeup (Castles et al., 1999) and the other because of 1 h of daily practice for 5 years. It is clarifying to maintain focus on exposing and explicating the measurement mechanism and not on distractions such as the myriad causes responsible for any specific attribute measure, be it temperature or reading ability.

In what we have termed theory-referenced measurement, instrument calibrations are provided by a construct theory and specifically not by data. For example, NexTemp thermometers and EdSphere™ reading tests^2^ are calibrated via theory. Person-fit statistics become not just checks on how similar a person's response data are to the reference group's data but on how well each person conforms to theoretical expectations. What is intended to be measured (e.g., temperature or reading ability) is made explicit with the theory-based instrument calibrations, and the fit statistics confirm or disconfirm whether the respective observed outcomes and their associated measures are consistent with theoretical expectations in the individual case (Smith, 2000).

One way in which causal Rasch models differ from descriptive Rasch models is in the pattern of counterfactual dependencies inherent in the former. Woodward (2003) referred to these dependencies as “w questions” (11): What if things had been different with either the attribute or the measurement mechanism? What value would the observed outcome take? We document these counterfactual dependencies by manipulating the attribute or the measurement mechanism or conjointly manipulating them both and seeing whether the expected score outcome is in fact observed. The trade-off property is a special kind of counterfactual dependence that can be used to test the quantitative status of constructs, as shown below.

## The measurement mechanism

Equation (2) is the familiar Rasch model for dichotomous data, which sets an observed outcome (raw score) equal to a sum of modeled probabilities. The observed outcome is the dependent variable and the measure (e.g., person parameter b) and instrument (e.g., item parameters d_*i*_) are independent variables. The concrete outcome (e.g., count correct on a reading test) is observed, whereas the measures and instrument parameters are not observed but can be estimated from the response data. In Equation (3), a mechanismic interpretation is imposed on the equation, the right-hand side (r.h.s.) variables are presumed to characterize the process that generates the observed outcome on the left-hand side (l.h.s.). An illustration of how such a mechanism can be exploited is given in Stone (2002). The item map for the Knox cube test (a test of short-term memory) revealed a 1 logit gap with no items. The specification equation was used to build an item that theory asserted would fill in the gap. Subsequent data analysis confirmed the theoretical prediction of the items scale location:

(1) $$\begin{array}{l} \text{Comprehension}=\text{Reading Ability}-\text{Text Complexity }\\ \text{ Conceptual Rasch Model} \end{array}$$

(2) $$\begin{array}{l} \text{Raw Score}=\sum_{i}\frac{e^{\left(b-\;d_{i}\right)}}{1+\;e^{\left(b-\;d_{i}\right)}}\\ \text{ Descriptive Rasch Model} \end{array}$$

(3) $$\begin{array}{l} \text{Raw Score}=\text{​}:\sum_{i}\frac{e^{\left(b-\;d_{i}\right)}}{1+\;e^{\left(b-\;d_{i}\right)}}\\ \text{ Causal Rasch Model} \end{array}$$

where raw score is the observed outcome, *b* is the attribute measure, and *d*_*i*_'s are mechanism calibrations. The observed outcome is thus, modeled as a sum of success probabilities. Typically, the item calibrations (*d*_*i*_'s) are assumed to be known and the measure parameter is iterated until the equality is realized (i.e., the sum of the modeled probabilities equals the observed outcome). How is this equality to be interpreted? Is something more happening than simply the algebra?

In an effort intended to clarify the practical value of the algebra, Freedman (1997) proposed three uses for regression equations like those above:

1. To describe or summarize a body of data,
2. To predict the l.h.s. from the r.h.s, and
3. To predict the l.h.s. after manipulation or intervention on one or more r.h.s. variables (measure parameter and/or mechanism parameters).

Description and summarization possess a reducing property in that they abstract away incidentals to focus on what matters in a given context. In a rectangular persons-by-items data matrix (with no missing data), there are N_*p*_ × N_*i*_ observations. Equations like those above summarize the data using only N_*p*_ + N_*i*_ − 1 independent parameters. Description and summarization are local in focus. The relevant concept is the extant data matrix with no attempt to answer questions that might arise in the application realm about “what if things were different.” Note that if interest centers only on the description and summarization of a specific data set, additional parameters can be added, as necessary, to account for or better describe the data (e.g., as in fitting a polynomial equation).

Prediction typically implies the use of the extant data to project into an as yet unobserved context/future in the application realm. For example, item calibrations from the extant data are used to compute a measure for a new person, or person parameters are used to predict how these persons will perform on a new set of items. Predictions like these rest on a set of invariance claims. New items and new persons are assumed to behave as persons and items behaved in the extant data set (Andrich, 1989). Rasch fit statistics (for persons and items) are available to test for certain violations of these assumptions of invariance (Smith, 2000).

To explain how an instrument works is to detail how it generates the count it produces (the observed outcome) and what characteristics of the measurement procedure affect that count. This kind of explanation is neither just statistical nor synonymous with prediction. Instead, the explanation entails prediction under intervention: If one wiggles this part of the mechanism, the observed outcome will imply a measure different by this amount. As noted by Hedström (2005), “Theories based on fictitious assumptions, even if they predict well, give incorrect answers to the question of why we observe what we observe” (108). Rasch models, absent a substantive theory capable of producing theory-based instrument calibrations, may predict how an instrument will perform with another subject sample (invariance) but can offer only speculation in answer to the question, “How does this instrument work?” Rasch models without theory are not predictive under intervention and thus, are not causal models.

In 1557, the Welshman Robert Recorde remarked that no two things could be more alike (i.e., more equivalent) than parallel lines, and thus, was born the equal sign, as in 3 + 4 = 7. We propose that the distinction between *descriptive* Rasch models and *causal* Rasch models should be signaled by the use of a 250-year-old symbol (=:) attributable to Euler (circa 1730) and exhumed by Pearl (1999), which denotes that interventions/manipulations on the r.h.s. of the equation *causes* a change to the l.h.s. of the equation (Pearl, 1999; Stenner et al., 2009). Allowable manipulations include changes to just reading ability, just text complexity, or conjointly to both. These equations purport to predict what will happen in the individual case (token causation) to the observed outcome (raw score, count correct, or percent correct) if allowable manipulations are made. Manipulations of reader ability and/or text complexity presume that these two variables (attributes of persons and text respectively), are well enough understood that manipulation is possible. Our recommended symbol also suggests that we adopt the more precise and explicit definition, as indicated above, to avoid the metaphysical implications of using causation in any narrative without an explicit understanding of what is implied. The symbol and definition reins in speculation surrounding the so-called swamp of language.

Cook and Campbell (1979) observed, “The paradigmatic assertion in causal relationships is that manipulation of a cause will result in the manipulation of an effect…. Causation implies that by varying one factor [variable] I can make another vary” (p. 36). Holland (1986) reduced this to an aphorism: “No causation without manipulation” and Freedman (1997) distinguished the merely descriptive from the causal:

Causal inference is different, because a change in the system is contemplated: for example, there will be an intervention. Descriptive statistics tell you about correlations that happen to hold in the data: causal models claim to tell you what will happen to Y if you change X. (116)

The descriptive Rasch model above, which employs a simple equality relating raw score to an exponentiated difference between ability (*b*) and item difficulty (*d*_*i*_), is a descriptive model (equation 2). Algebraic manipulation of the ability parameter or item difficulty parameter can be evaluated for consequences to the raw score, but this says nothing about what would result following an experimental manipulation of *b* or *d* or a conjoint intervention on both simultaneously. Following Woodward (2003), we require causal Rasch models to be modular in the sense that it is possible to intervene on (manipulate) one variable in the equation on the right side without affecting another right-hand variable. Specifically, a manipulation of text complexity (e.g., choosing a more difficult text) should not alter reader ability and vice versa. A causal Rasch model should expose the mechanism that transmits variation in the attribute to the observed outcome: “One would also like to have more detailed information about just which interventions on X [r.h.s. of the equation] will change Y [the score or comprehension rate] and in what circumstances and exactly how they will change Y” (Woodward, 2003, p. 66). Reflecting on the distinction between a descriptive use and a causal use of a Rasch equation, asymmetry is apparent. A causal model can always be used for merely descriptive purposes even though it can do more, whereas a descriptive (e.g., correlational) model can offer no predictions about what would happen if right-side variables were manipulated as in equation (3).

It is often argued in garden-variety Rasch applications that the best evidence that an instrument is doing what it is supposed to do is data fit to the Rasch model, which implies that the observed outcome (e.g., count correct on a reading test) is a sufficient statistic for the attribute measure and so exhausts the information in the data about the attribute measure. Conditional on the difference between attribute measure (e.g., temperature) and measurement mechanism (amount of additive in a NexTemp cavity) the residuals taken over persons and taken within person over time are uncorrelated. Unfortunately, nothing in the fact that data fit the model licenses the conclusion that what is being measured is “temperature” or “reading ability” (Maraun, 1998). Typical so-called science tests are often poorly disguised reading tests. More than good fit of data to the model are needed to decide among competing claims regarding what attribute an instrument measures. We speculate that this fact is poorly understood because it is believed that conditioning on a variable is the same thing as intervening to fix the value of that variable. The former is a statistical manipulation and the latter is an experimental manipulation: “When one conditions, one takes as given the probability distribution. When one intervenes, one changes the probability distribution” (Hausman, 1998, p. 233). This is an important difference between descriptive Rasch models and causal Rasch models.

*Measurement mechanism* is the name given to just those manipulable features of the instrument that cause invariant observed outcomes for objects of measurement that possess identical measures. A measurement mechanism explains by opening the black box and showing the cogs and wheels of the instrument's internal machinery. A measurement mechanism provides a continuous and contiguous chain of causal links between the encounter of the object of measurement and instrument and the resulting observed outcome (Elster, 1989). We say that the observed outcome (e.g., raw score) is explained by explicating the mechanism by which those outcomes are obtained. In this view, to respond with a recitation of the Rasch equation for converting counts into measures, to reference a person by item map, to describe the directions given to the test-taker, to describe an item-writing protocol, or simply to repeat the construct label more slowly and loudly (e.g., extroversion), provide non-answers to the question, “How does this instrument work?”

Measurement mechanisms as theoretical claims, made explicit as specification equations, make point predictions under intervention: When one changes (via manipulation or intervention) either the object measure (e.g., reader experiences growth over a year) or measurement mechanism (e.g., increase text complexity measure by 200 L), the result will be a predictable change in the observed outcome. Notice how this process is crucially different from the prediction of the change in the observed outcome based on the selection of another, previously calibrated instrument with known instrument calibrations. Selection is not intervention in the sense used here. Sampling from banks of previously calibrated items is likely to be completely atheoretical, relying, as it does, on empirically calibrated items/instruments. In contrast, to modify the measurement mechanism requires intimate knowledge of how the instrument works. A theoretical psychometrics is characterized by the aphorism “test the predictions, never the postulates” (Jasso, 1988, p. 4), whereas theory-referenced measurement, with its emphasis on measurement mechanisms, says test the postulates, never the predictions. Those who fail to appreciate this distinction will confuse invariant predictors with genuine causes of observed outcomes.

We assert that a Rasch model combined with a substantive theory embodied in a specification equation provides a more or less complete explanation of how a measurement instrument works (Stenner et al., 1983). A Rasch model in the absence of a specified measurement mechanism is merely a probability model; a probability model absent a theory may be useful for Freedman's (1) and (2), whereas a Rasch model in which instrument calibrations come from a substantive theory that specifies how the instrument works is a causal model; that is, it enables prediction after intervention [i.e., Freedman's (3)].

## Distinguishing features of causal rasch models

Admittedly, the measurement model we have proposed for the human sciences mimics key features of physical science measurement theory and practice (Bond and Fox, 2007). Below we highlight several such features.

First, the model is individual-centered. The focus is on explaining variation within person over time. Much has been written about the disadvantages of studying between-person variation with the intent to understand within-person causal mechanisms (Molenaar, 2004; Molenaar and Newell, 2010). Molenaar proved that only under severely restrictive and generally untenable conditions can such cross-level inferences be sustained. In general, in the human sciences, it is necessary to build and test individual-centered models and not rely on variable- or group-centered models (with attendant focus on between person variation) to inform one's understanding of causal mechanisms. Causal Rasch models are individually centered measurement models. The measurement mechanism that transmits variation in the attribute (within-person over time) to the observed outcome (count correct on a reading test) is hypothesized to function the same way for every person (the second ergodicity condition of homogeneity; Molenaar and Newell, 2010).

Second, in this framework, the measurement mechanism is well specified and can be manipulated to produce predictable changes in observed outcomes (e.g., percentage correct). For purposes of measurement theory, a sophisticated philosophy of causal inference is not necessary. For example, questions about the role of human agency in the intervention- and manipulation-based accounts of causal inference are not troublesome here. All that is meant by the claim that the r.h.s. of equation (3) causes the l.h.s. is that experimental manipulation of each r.h.s. variable will have a predictable consequence for the observed outcome (expected raw score). Stated more generally, what is meant by x causes y is that an intervention on x yields a predictable change in y. The specification equation used to calibrate instruments/items is a recipe for altering just those features of the instrument/items that are causally implicated in the observed outcome. As noted, we term this collection of causally relevant instrument features the measurement mechanism, which transmits variation in the attribute (e.g., temperature, reading ability) to the observed outcome (number of cavities that turn black or number of reading items answered correctly). Two additional applications of the specification equation are: (a) the maintenance of the unit of measurement independent of any particular instrument or collection of instruments, and (b) bringing non-test behaviors (reading a Harry Potter novel, 980 L) into the measurement frame of reference (Stenner and Burdick, 2011).

Third, item parameters are supplied by substantive theory and thus, person-parameter estimates are generated without reference to or use of any data on other persons or populations. When data fit a Rasch model, a consequence is that item parameters are estimated independent of person parameters and person parameters are estimated independent of item parameters (Rasch, 1960; Andrich, 1989, 2002). In a causal Rasch model, item/instrument calibrations are supplied by a substantive theory and associated specification equation. In the former, the separation is statistical; in the latter it is experimental. Karabatsos (2001) commented on the impossibility of complete separation when the same response data are used to estimate both person measures and item calibrations. Effects of the examinee population are completely eliminated from consideration in the estimation of an individual's person parameter and, thus, no information on other persons is needed because the item/instrument calibrations come from theory.

Fourth, the quantitative hypothesis (Michell, 1999) can be experimentally tested by evaluating the trade-off property for the individual case. A change in the reader parameter can be offset or traded off for a compensating change in text complexity to hold comprehension constant. The trade off is not just about the algebra in equations (1–3). It is about the consequences of simultaneous intervention on the attribute (reader ability) and measurement mechanism (text complexity).

Finally, we conjecture that successful point predictions under intervention necessitate quantitative predictors and outcomes. Concretely, if an intervention on the measurement mechanism (e.g., increase the text complexity of a reading passage by 250 L) results in an accurate prediction of the observed outcome (e.g., how many reading items the reader will answer correctly), and if this process of offsets can be successfully repeated up and down the scale, then text complexity, reader ability, and comprehension (success rate) are quantitative attributes of the text, person, and reader/text encounter, respectively. When text complexity is measured on an ordinal scale, successful point predictions about counts correct based on a reader/text difference are impossible to make. Specifically, successful prediction from differences requires that what is being differenced has the same meaning up and down the respective scales. Differences on an ordinal scale are not meaningful (will lead to inconsistent predictions) precisely because *one more* means something different depending on the location of the text or reader on the scale.

The algebra in equation (2) dictates that a change in reader ability can be traded off for an equal change in text complexity to hold comprehension constant. However, to test the quantitative hypothesis requires more than the algebraic equivalence in a Rasch model. Rather, what is required is an experimental intervention/manipulation on either reader ability or text complexity or a conjoint intervention on both simultaneously that yields a successful prediction on the resultant observed outcome (count correct). We maintain that when manipulations of the type just described are introduced for individual reader/text encounters and model predictions are consistent with what is observed, the quantitative hypothesis is sustained.

We emphasize that the above account is individual-centered as opposed to group-centered. The Lexile Framework for Reading purports to provide a causal model for one aspect of what transpires when a reader reads a text. Nothing in the model precludes averaging over readers and texts to summarize evidence for the quantitative hypothesis, but the model can be tested at the individual level. These individual-level tests follow through from Rasch's (1960, pp. 110–115) care in structuring his models in the same mathematical form as Maxwell's analysis of force, mass, and acceleration. By deliberately requiring models of this form, Rasch employs Maxwell's own method of analogy (Turner, 1955; Nersessian, 2002) and enables us to apply it, as Maxwell did, in setting up individual-level tests of hypotheses about potential causal relations among the model parameters (Fisher, 2010).

Maxwell's use of the method of analogy in developing electromagnetic theory has been shown to extend and focus everyday thinking processes into generic scientific model-based reasoning processes (Nersessian, 2006, 2008). In these reasoning processes, formal and structural analogies do not in any way imply content-based analogies. Thus, Rasch's analogy from masses and forces to persons and test items has no psychophysical connotations implying a role for mass and force in the way people respond to assessment questions. The point here extends beyond the present concerns to concept formation in science generally: neither Rasch's models nor Newton's laws are conclusions drawn from observations, being based as they are in reasoning from geometry, other equations, and information (see Crease, 2004a,b for lists of similar mathematical laws).

The analogy is purely formal, and could involve any number of similarly structured relations from a wide variety of other domains in which lawful causal patterns are found, such as chemistry or genetics. No special value beyond their familiarity is to be inferred from the choice of physical constructs in the analogies made. That said, just as pressure and volume can be traded off to hold temperature constant or mass and volume can be traded off to hold density constant, so can reader ability and text complexity be traded off to hold comprehension constant. Michell (1999) made the following remarks on this point: “Identifying ratios directly via trade-offs results in the identification of multiplicative laws between quantitative attributes. This fact connects the theory of conjoint measurement with what Campbell called derived measurement” (204). See Kyngdon (2008) for a particularly clear discussion of this connection.

Garden-variety Rasch models and IRT models are in their application purely descriptive. They become causal and law-like when manipulations of the putative quantitative attributes produce changes (or not) in the observed outcomes that are consistent with model predictions. If a fourth-grade reader grows 100 L in reading ability in 1 year and the text complexity of the student's fifth-grade science textbook also increases by 100 L over the fourth-grade textbook, then the forecasted comprehension rate (whether 60, 70, or 90%) that the reader will enjoy in fifth grade science remains unchanged from that experienced in fourth grade. We offer without proof that only if reader ability and text complexity are quantitative attributes will experimental findings coincide with model predictions such as these. We have tested several thousand students' comprehension of 719 articles that averaged 1150 words in length. Total reading time was 9794 h and the total number of unique machine-generated comprehension items was 1,349,608. The theory-based expectation was 74.53% correct and the observed was 74.27% correct.

A measurement instrument comprises two kinds of features: radicals and incidentals (Irvine and Kyllonen, 2002). The *radicals* are those features that transmit variation in the attribute to the observed outcome. Radicals are tunable and when intervened upon, will change what is observed (count correct or number of cavities turning black). *Incidentals* include all features of the measurement instrument that if manipulated will not change the observed outcome. To change sentence length and vocabulary level will alter a text's complexity and will change count correct on an embedded reading test (observed outcome), whereas to change the font style will not. Stenner et al. (1983) and Stone and Wright (1983) proposed that radicals could be organized into a specification equation and could be used to provide theory-based calibrations for items, instruments, and ensembles. A powerful demonstration that a measurement process is under control is provided by a tradeoff between an intervention on the attribute measure (e.g., reading ability) for an intervention on the measurement mechanism/specification equation (e.g., text complexity) to hold the observed outcome (relative raw score, percentage correct) constant. Only when an instrument can be tuned to produce a desired change in the observed outcome when holding the attribute measure constant is the measurement mechanism well understood. This is, of course, the feature that enables the manufacture of large numbers of instruments that share the same correspondence table linking observed outcome to attribute measure. We call such instruments *strictly parallel*.

We maintain that when data fit a descriptive Rasch model, there is an important sense in which we remain dissatisfied. The source of this dissatisfaction resides in the fact that scientists eschew theories and models that contain many “free parameters… the values of which are not determined by the theory itself but rather must, as it is commonly expressed, ‘be put in by hand’—introduced with no other rationale than they are required by the data” (Woodward, 1989, p. 364). In descriptive Rasch models (and all IRT models), item and instrument calibrations are estimated from data, whereas in a causal Rasch model, the item, ensemble, or instrument calibrations are provided by theory, thus, dramatically reducing the free parameters in the model and not so coincidentally reducing the sense of arbitrariness.

We cannot overstate the importance of describing the measurement mechanism when explaining why a particular scored outcome was observed or how it came to be. If a Rasch or IRT study is submitted for publication and it makes no attempt to explicate the mechanism or active ingredients that transmit variation in the attribute to the observed outcome, then a truth-in-advertising disclaimer such as, “unfortunately, no mechanism is known to underlay the Rasch equation that we use,” should accompany the report.

The simple fact that data fit a Rasch model where the dependent variable is count correct on a test and the predictor is a difference between a person parameter and an instrument parameter does not elicit understanding of the mechanism at work and thus, does not explain: “Because to explain is to exhibit or assume a (lawful) mechanism. This is the process—whether causal, random, or mixed that makes the system work the way it does…. This kind of explanation is usually called mechanistic. I prefer to call it mechanismic, because most mechanisms are not mechanical” (Bunge, 2004, p. 203). Without a mechanism (modeled as a specification equation), a Rasch model is unsatisfyingly functional and descriptive rather than mechanismic and explanatory. If editors embraced this position, attention might shift to explications of the mechanisms that underlie the human and social science instrumentarium.

The role of causal inference in human science measurement theory has been underdeveloped in part because causal inference is philosophically complex and more specifically the so-called mechanismic interpretation of measurement (Stenner et al., 2009) has lived in the shadow of sampling-based frameworks such as facet theory (Guttman, 1971), generalizability theory (Brennan, 2011), true score theory (Lord and Novick, 1968) and behavior domain theory (McDonald, 2009).

## Illustrating the tradeoff property

In adopting the tradeoff property as a useful test for quantity in the human and social sciences, we reasoned that the symmetry of a Rasch model lends itself to thought about offsetting manipulations on the r.h.s. of equation (3) producing no change to the l.h.s. Concretely, a manipulation that increases text complexity by 200 L, if offset by an increase of 200 L in reader ability, should yield no change in observed comprehension rate. So, offsetting manipulations in two distinct attributes, text complexity and reader ability, can be experimentally shown to hold constant a third attribute (comprehension). If this tradeoff property holds up and down the scales for all three variables in the Lexile equation, the attributes (text complexity, reader ability, and comprehension) we conjecture must be quantitative attributes of text, reader, and the reader-text encounter, respectively. As we have seen, a particularly attractive feature of this approach to testing the quantitative hypothesis is that one can perform the test within-person with no reference to any between-person relations. Specifically, over a 13-year period (grades K-12), a reader is growing in reading ability and the computer trades off growth for new texts that have just the right amount of added complexity to hold the comprehension rate constant. Over the 13 years, the reader may read thousands of articles and millions of words, but the whole history can be summarized by the expected minus observed count correct. We assert without proof that only if text complexity, reader ability, and comprehension are quantitative, will we consistently observe a close correspondence between expected (under the theory) and observed count correct on machine-generated cloze items. Because one always wants the quantitative hypothesis to be sustained within-person over time, it seems best to test the hypothesis at the individual level and not resort to cross-level inferences (e.g., attempts to infer from between-person relationships something about within-person processes), which often find dubious rationales (Molenaar and Newell, 2010).

**Figure 1:**
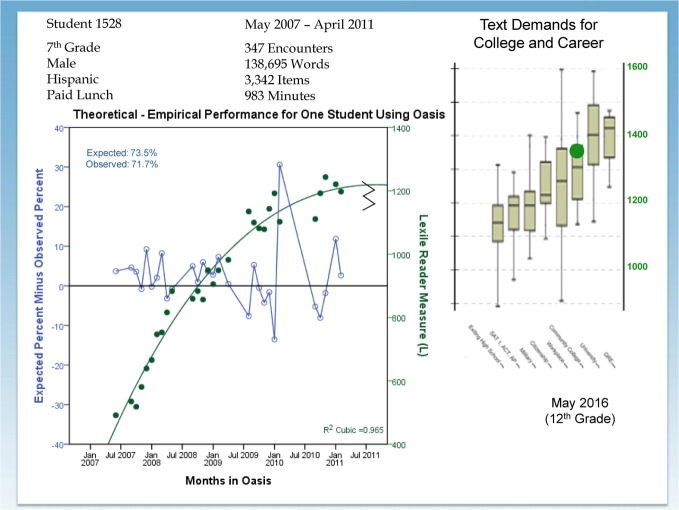
**Growth in reading ability relative to the reading demands of adulthood**.

Figure 1 is an individual-centered growth trajectory for reading ability denominated in Lexiles. Student 1528 is a seventh grade male who read 347 articles (138,695 words) between May 2007 and April 2011. Each solid dot corresponds to a monthly average Lexile measure. The growth trajectory fits the data quite well, and this young man is forecasted (big dot on the far right of the figure) to be a college-ready reader when he graduates from high school. The open dots distributed around O on the horizontal axis are the expected performance minus observed performance for each month. Expected performance is computed using the Rasch model and inputs for text complexity and the reader's ability measure. Given these inputs, the apparatus forecasts a percentage correct. The observed performance is the observed percentage correct for the month. The difference between what the substantive theory (Lexile Reading Framework) in cooperation with the Rasch model expects and what is actually observed is plotted by month. The upper left-hand corner of the graphic summarizes the expected percentage correct (73.5%) and observed percentage correct (71.7%) across the 3342 items taken by this reader during 4 years. What may not be immediately obvious is that the apparatus is dynamically matching text complexity to the developing reader's ability to hold comprehension (percentage correct) at 75%. So, this graphic describes a within-person (intra-individual) test of the quantitative hypothesis: Can the apparatus trade off a change in reader ability for a change in text complexity to hold constant the success rate (comprehension)?

When a wide range of data fit a causal Rasch model, does the model assume the status of a law such as F = MA? Although the lines of demarcation between invariant generalizations and laws are difficult to draw, one feature seems paramount. How phenomena relate to one another must be independent of the particular mechanism used to measure each phenomenon. This independence presumes that multiple mechanisms exist for measuring each phenomenon that figures in the law. F = MA would not be a law if it mattered how exactly mass were measured (what kind of weighing apparatus was employed) and similarly for acceleration and force. In fact, the very claim for existence of a phenomenon (reader ability or text complexity) depends upon the fact that *different calibrated mechanisms return the same amounts.* Without demonstrated invariance of a phenomenon over measurement mechanisms, a counterclaim remains open that the hypothesized phenomenon is artifactually dependent on one particular instrument or measurement mechanism. We conjecture that laws are laws, in part, because they are invariant under changes in measurement mechanism(s).

So, what does it mean to claim that the r.h.s. of equation (3) is causal on the l.h.s. and thus, that the causal operator (=:) should be used in place of (=). Borrowing from Woodward (2003), we mean that an intervention or manipulation of the measurement mechanism and/or the attribute measure changes the observed outcome. The mathematical model explains how observed outcomes (counts correct on a reading test) are dependent on the measurement mechanism (text complexity and task type) and measured attribute (reader ability). The substantive theory specifies precisely what kinds of interventions on the object of measurement and measurement mechanism will change the observed outcome and, by omission, what interventions should have no effect on the observed outcome. In this sense, measurement as envisioned in the standard measurement model is about manipulation and control and not about correlation, description, and classification.

**Figure 2:**
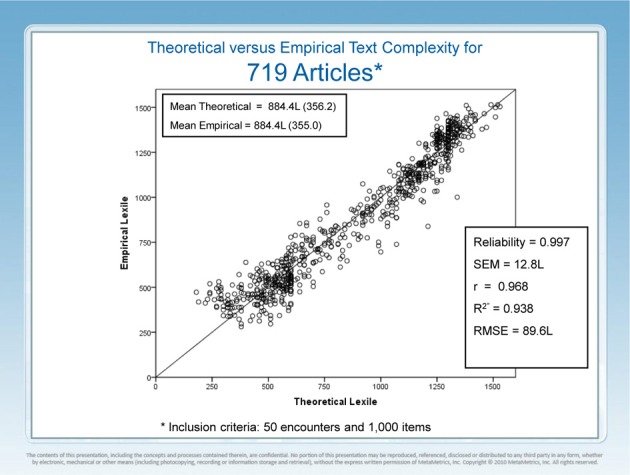
**Predicted vs. observed text complexity measures**.

Figure 2 presents the results of a 5-year study of the relationship between theoretical text complexity as provided by a computer based text complexity engine (Lexile Analyzer) and empirical text complexity as provided by the EdSphere™ platform. The Lexile Analyzer computes the semantic demand of a text proxied by the log transformed frequency of each word's appearance in a multibillion word corpus of published text and the syntactic demand of each text proxied by log transformed mean sentence length. The text preprocessing, what constitutes a *word* and what constitutes a sentence ending, involves thousands of lines of code. Modern computing enables the measurement of Tolstoy's *War and Peace* in a couple of seconds. The Lexile Analyzer has taken 25 years to build and optimize and is freely available for non-commercial use (Lexile.com).

The EdSphere™ platform enables students to select articles of their choosing from a vast range of content. Selected articles are targeted to ± 100 L of each student's developing reading ability. Thus, as students' reading ability grows, the machine adjusts the text complexity of the articles from which the student chooses the next reading. The target success rate is 75%. The machine generates a reading comprehension item on the fly about every 70 words such that two students sitting side by side at computers and reading the same article will respond to different items.

Because only very rarely will more than one student take any particular item, a new kind of Rasch model was needed. The *ensemble* Rasch model exploits the raw score sufficiency property of Rasch models to convert counts correct on unique sets of items into Lexiles (Stenner et al., 2006; Lattanzio et al., 2012). The text complexity measure for the present article is 1480 L, which also is the mean difficulty of all the allowable cloze reading items that could be machine constructed for this article. Given this ensemble mean and a theory-specified ensemble variance, counts correct can be converted into a Lexile measure by sampling item calibrations from the ensemble and using these item calibrations to solve the Rasch equation. The key intuition that makes this possible is that it does not matter which particular sampled item calibrations are attached to which particular machine-generated items because a consequence of raw score sufficiency is that there is no information in the pattern of rights and wrongs about the reader parameter; thus, it does not matter how the sampled item calibrations are attached to individual items.

The 719 articles in this study were chosen because they were the first articles to meet the dual requirements of at least 50 readers and at least 1000 item responses. Well-estimated reader measures were available prior to an encounter between an article and a reader. The ensemble Rasch equation (not given) was rearranged to solve for text complexity given a count correct, a reader ability, and an ensemble variance. Thus, each of the 719 articles had a theoretical text complexity from the Lexile Analyzer and an empirical text complexity from EdSphere™. The correlation between theoretical text complexity and empirical text complexity is *r* = 0.968 (*r*^2^ = 0.938). Reliability of the empirical text complexity measures is *r*_*tt*_ = 0.997 and the *RMSE* = 89.6 L. The RMSE is the square root of the average squared difference between the empirical and theoretical text complexities. Work has been ongoing for two decades to isolate new linguistic variables that might account for the 6% of uncontrolled variance. More recent work has shifted to a focus on possible artifacts (measurement error, range restriction, text preprocessor variation, and task specificity) that might account for the small remaining uncontrolled variation.

When data fit a descriptive Rasch model, relative differences between objects of measurement on the attribute of interest are independent of the instrument. When data fit a causal Rasch model in which instrument calibrations (item difficulty estimates) come from theory, then absolute measures are independent of instrument. Rasch called the former *specific objectivity*, and we called the latter *general objectivity* (Stenner, 1994). Both the temperature and reading attributes used as illustrations in this paper have available, well-developed, generally objective measurement systems. Equation (3) is an individual-centered measurement model (token causal model) and can be distinguished from SEM models and much philosophical discussion on causation that focuses on type causal models.

There is an unfortunate tendency in reading research and psychometrics to equate changes in measurement mechanism (e.g., task type) with change in the attribute being measured. For example, it is asserted that multiple-choice tests of reading must measure something other than short-answer, constructed responses because the former measure superficial, fact-based understanding and the latter measure higher level inferences. Thirty years ago, reading part scores were reported for subtests that mistakenly were thought to measure something else because the questions focused on different levels of Bloom's taxonomy. The idea that a reader constructs a meaning model and that all reading items interrogate that meaning model (Kintsch, 1974) and that different item types might measure the same attribute (reader ability) but with different added easiness or difficulty relative to some standard task type was a foreign notion. Adoption of the Lexile Reading Framework was resisted in part because it appeared to be too simple. The assertion that reading ability, like temperature, is a unidimensional attribute that can be measured with many mechanisms that appear different has been slow to take hold. That said, some measurement mechanisms may confound two or more attributes. Mercury in a tube thermometer without a top confounds the measurement of temperature with the measurement of atmospheric pressure. To seal the top of the thermometer eliminates the confound. Similarly, to ask readers to summarize in writing what they have just read may confound reading ability and writing ability. Both attributes of language facility are important, but for purposes of measurement, it is important to have mechanisms that transmit only one kind of variation and not a confounded mixture of two or more distinct kinds of variation.

## Instrument validity and instrument validation

Consider the following statements about validity and ponder the theoretical and practical implications that might follow for the measurement, respectively, of temperature and reading ability:

Validation of an instrument calls for an integration of many types of evidence. The varieties of investigation are not alternatives any one of which would be adequate. The investigations supplement one another…. For purposes of exposition, it is necessary to subdivide *what in the end must be a comprehensive, integrated evaluation of the test*. (Cronbach, 1971, 445; emphasis in original)

[Validity is] an integrated evaluative judgment of the degree to which empirical evidence and theoretical rationales support the *adequacy* and *appropriateness* of *inferences* and *actions* based on test scores or other modes of assessment. (Messick, 1989, 13; emphasis in original)

Validation involves the evaluation of the proposed interpretations and uses of measurements. The interpretive argument provides an explicit statement of the inferences and assumptions inherent in the proposed interpretations and uses. The validity argument provides an evaluation of the coherence of the interpretive argument and of the plausibility of its inferences and assumptions. It is not the test that is validated and it is not the test scores that are validated. It is the claims and decisions based on the test results that are validated. Therefore, for validation to go forward, it is necessary that the proposed interpretations and uses be clearly stated. (Kane, 2006, 59–60)

So, from the above, validity calls for synthesis or integration of diverse sources of evidence. It is useful, for purposes of exposition, to group these sources. Not mentioned in the quotes above but described elsewhere are dozens of validity categories [face, criterion, concurrent, convergent, discriminant, predictive, construct, and 200 more documented by Shaw and Newton (2012) and Newton and Shaw (2012)]. For Cronbach, Messick, and Kane validity does not apply to the instrument (thermometer or reading test) nor just to the numbers produced by these instruments but rather to the claims and decisions that are made by users based on these numbers.

Even a casual reading of the history of science reveals a stunning disconnect between the quotations above and how instruments are invented, improved, and justified in the physical sciences. What holds center stage in physical science measurement is substantive theory (Maraun, 1998; Sherry, 2011) followed by intense focus on precision of measurement and to a lesser degree by an imperative for readable technologies (directly perceivable quantities for the latent variable being measured). Great care is taken in physics, for example, to separate what is being measured by an instrument from the uses to which measurements might be put and from the consequences of these uses. It is not that these consequential considerations are unimportant but that they have nothing to do with what an instrument measures. Validity, for us, is all about what an instrument measures.

Contrast the quotes from Cronbach, Messick, and Kane with those of Stenner et al. (1983) and Borsboom (2005):

The notion that a test is valid if it measures what it purports to measure implies that we have some independent means of making this determination. Of course, we usually do not have an independent standard; consequently, validation efforts devolve into circular reasoning where the circle generally possesses an uncomfortably small circumference. Take, for example, Nunnally's (1978) statement, “A measuring instrument is valid if it does what it is intended to do” (p. 86). How are we to represent intention independent of the test itself? In the past, educators and psychologists have been content to represent intentions very loosely, in many cases letting the construct label and its fuzzy connotations reconstruct the intentions of the test developers. Unfortunately, when intentions are loosely formulated, it is difficult to compare attainment with intention. This is the essence of the problem faced by classical approaches to validity. Until intentions can be stated in such a way that attainment can be explicitly tested, efforts to assess the adequacy of a measurement procedure will be necessarily characterized by post hoc procedures and interpretations. (Stenner et al., 1983, 31)

Thus, validity theory has gradually come to treat every important test-related issue as relevant to the validity concept, and aims to integrate all these issues under a single header. In doing so, however, the theory fails to serve either the theoretically oriented psychologist or the practically inclined tester; the theoretically oriented are likely to get lost in the intricate subtleties of validity theory, while the practically oriented are unlikely to derive a workable conceptual scheme with practical implications from it. (Borsboom, 2005, 150)

If we follow this thread, where does it lead? First, let's observe that the past 400 years of successful physical science have managed with only the rudiments of a formal measurement theory. Progress in every physical science measurement armament has been realized from improved theory and improved engineering in the service of ever-increasing precision of measurement, and that is about it. Human science measurement, in contrast, has expended virtually no energy on substantive theory development, nor on increasing, say by an order of magnitude, the precision of ability, personality, or attitude measurement. Rather, we psychometricians expend much energy on mathematizing everything we do in a kind of caricature of physical science measurement without grasping the significance of just what physical science is accomplishing (Maraun, 1998; Sherry, 2011).

At present, much of the human science is too frequently actuarially driven rather than theory-driven. Consider the multitrillion dollar insurance industry as an analogy. The foundations of the industry are highly mathematized and predictions in the aggregate are highly precise, but there is no body of increasingly integrated substantive theory. Actuarial science has given us some wonderfully useful mathematics and in turn has found uses for some elegant pure mathematics. This sophisticated mathematization operates in the service of a simple criterion: prediction of how a population (humans, container ships, orange trees) will fare next year or next decade. As useful as this may be within the limits of the insurance industry's interests, theory-driven science optimizes a completely different kind of criterion.

The notion that validity is about the conformance of what an instrument actually measures to what the developers intended or what they *purport* to measure is vacuous unless *intent* can be independently formulated as, for example, in a specification equation. Too often in practice the instrument is offered as evidence both of intent and attainment of that intent. The specification equation breaks the relationship between intention and attainment by independently formalizing intent as an equation that can produce item calibrations or ensemble means that are in close correspondence with empirical estimates. Instruments are designed to detect variation of a kind. The specification equation specifies the kind and provides a means by which to test whether the variation detected by the instrument aligns with that specified by the construct theory:

The construct specification equation affords a test of fit between instrument-generated observations and theory. Failure of a theory to account for variation in a set of empirical item scale values invalidates the instrument as an operationalization of the construct theory and limits the applicability of that theory. Competing construct theories and associated specification equations may be suggested to account for observed regularity in item scale values. Which construct theory emerges (for the time) victorious, depends upon essentially the same norms of validation that govern theory evaluation in other sciences. (Stenner et al., 1983, 307)

Everything important to instrument justification cannot be subsumed under the validity banner. Validity is not constituted by a bucket of correlations. It was these realizations among other deficiencies in the concept that led us to reformulate validity as a correspondence between intent and attainment where intent is formalized as a specification equation. Borsboom (2005) said it best:

The argument to be presented is exceedingly simple, so simple, in fact, that it articulates an account of validity that may seem almost trivial. It is this: if something does not exist, then one cannot measure it. If it exists, but does not causally produce variations in the outcomes of the measurement procedure, then one is either measuring nothing at all or something different altogether. Thus, a test is valid for measuring an attribute if and only if (a) the attribute exists, and (b) variations in the attribute causally produce variations in the outcomes of the measurement procedure.

The fact that the crucial ingredient of validity involves the causal effect of an attribute on the test scores implies that the locus of evidence for validity lies in the processes that convey this effect. This means that tables of correlations between test scores and other measures cannot provide more than circumstantial evidence for validity. What needs to be tested is not a theory about the relation between the attribute measured and other attributes, but a theory of response behavior. Somewhere in the chain of events that occurs between item administration and item response, the measured attribute must play a causal role in determining what value the observed outcomes will take; otherwise the test cannot be valid for measuring the attribute. Importantly, this implies that the problem of validity cannot be solved by psychometric techniques or models alone. On the contrary, it must be addressed by substantive theory. (151)

It is often difficult to conduct experimental manipulation (in the short term) of the latent variable (e.g., reader ability) of interest. We can and do, however, exploit the symmetry in the Rasch model and experimentally manipulate the items (e.g., text complexity) to produce the observed outcome (e.g., success rate or comprehension) expected under the construct theory. Manipulation of the text by the use of the specification equation results in changes consistent with theoretical expectations. The specification equation is one vehicle by which to introduce substantive theory into a psychometric model. The potential of this approach can perhaps be grasped most easily by setting up an equivalence relation. Suppose one wants to set the observed outcome (e.g., count correct on a reading test) equal for two readers who differ in reading ability. One could use the specification equation to build and calibrate items for the better reader that are just enough more difficult than the easier items given to the less able reader to cancel the way the first reader's ability surpasses that of the second reader. This trade off, or cancellation property, characterizes additive conjoint measurement frameworks (Michell, 2005; Kyngdon, 2008). In the Lexile Framework for Reading, a difference in reader ability of 200 L can be traded off for a difference in text readability of 200 L to hold constant the comprehension rate (Burdick et al., 2006). Relying only on substantive theory, the specification equation enables these causal manipulations.

In summary, validity is a straightforward concept with a specific meaning; it is not an amalgamation of dozens of kinds of evidence. Validity does not reduce to the scientific method and there is nothing in need of unification in the concept. The usefulness of measures and the consequences (intended and unintended) of their use, although important, have nothing to do with validity. In fact, “validity is not a judgment at all. It is the property being judged” (Borsboom, 2005, p. 154). When asserting that an instrument is a valid measure of an attribute, one makes an ontological claim that the attribute exists (a realist stance), that the instrument detects variation in the attribute, and that experimental manipulation of the attribute or the instrument (mechanism) will cause theoretically expected changes in the observed outcome (count correct).

If a measurement instrument's primary purpose is to detect variation of a kind then the paramount validity question is what causes the variation detected by the instrument. The specification equation provides an answer to this question by formalizing what it means to move up and down a scale for an attribute.

How well empirical item difficulties or ensemble means align with theoretical calibrations is a matter of degree (Stenner et al., 2006). Alternative construct theories and their attendant formalizations as specification equations compete in accounting for the variation detected by an instrument. As such, we adopt a causal rather than a correlational view of validity. We accentuate the role of substantive theory in our approach and have advanced the term *theory-referenced measurement* to label the method (Stenner et al., 2009).

## Conclusion

Causal Rasch models expose the mechanism that transmits attribute variation to the observed outcome. Specification equations are one useful way to expose and express a mechanism's action. Although a specification begins its life as a local descriptive account of specifically objective item/instrument variation on a single form of a test, it can evolve into a universal causal account of the behavior of items/instruments previously used to measure an attribute and all items and instruments that might yet be manufactured. The specification equation bridges the here and now of the test in hand and the infinity of possible instruments that can be engineered and manufactured for the measurement of an attribute. The equation provides not only a specification for instrument manufacture but also yields the calibrations that can be used to convert counts into quantities.

So, an early use of the specification equation is to operationalize Rasch's (1960) notion of a frame of reference in a way that extends the frame beyond the specific objectivity obtained in the context of one or two tests for an attribute to an indefinitely large collection of actual and imagined instruments. Theory-based instrument calibration eliminates the need to use data both to calibrate instruments and to measure persons. The payoff of using theory instead of data to calibrate instruments is large and immediate. When data fit a Rasch model, differences among person measures are free of dependencies on other facets of the measurement context (i.e., differences are specifically objective). When data fit a causal Rasch model, absolute person measures are free of the conditions of measurement (items, occasions, contexts) and thus, absolute measures are objective. Under a causal Rasch model, attribute measures (temperature or reading ability) are individually centered statistics precisely because no reference to other person(s) data figures in their estimation.

Applications of descriptive Rasch models have (over the last 50 years) identified thousands of data sets that fit. For only a handful of these has there been an attempt to explicate the mechanism that transmits attribute variation to the observed outcome. We do not, however, want to miss the real possibility that there exist mechanisms for a non-negligible subset of these applications and that some mechanisms will explain observed outcomes across multiple applications. A starting point for this search might begin with the question, “How does this instrument work?” What are the characteristics of the items/instrument that, if experimentally manipulated, could be expected to change the observed outcome? Even tentative answers to these questions will move psychometrics into a closer alignment with the standard measurement model (Fisher and Stenner, 2011, 2012).

There has been a groundswell of negativity regarding the potential of psychology to realize *measurement* as that term is understood in the physical sciences (Cliff, 1992; Schönemann, 1994; Michell, 2000; Barrett, 2008; Grice, 2011). More recently, Trendler (2009) concluded, “Psychological phenomena are not sufficiently manageable. That is, they are neither manipulable nor are they controllable to the extent necessary for an empirically meaningful application of measurement theory. Hence they are not measurable” (592). For Trendler, psychology cannot have measurement because it cannot manipulate and control its constructs. This paper offers a demonstration that psychological measurement might be possible. Furthermore, a roadmap is presented for the realization of measurement for those attributes that, like early conceptions of temperature, are today merely ordinal.

### Conflict of interest statement

Two of the authors (Stenner and Burdick) are employees of MetaMetrics, Inc., which developed and markets the Lexile Framework for Reading, an approach to unifying reading measurement that incorporates the kinds of causal mechanisms described in this article. Fisher is a paid consultant with MetaMetrics. Stone is the author of a commercially available instrument, the Knox Cube Test (Revised), which also incorporates the kinds of causal mechanisms described in the article.

## Footnotes

^1^“The NexTemp thermometer is a thin, flexible, paddle-shaped plastic strip containing multiple cavities. In the Fahrenheit version, the 45 cavities are arranged in a double matrix at the functioning end of the unit. The columns are spaced 0.2°F intervals covering the range of 96.0°F to 104.8°F…. Each cavity contains a chemical composition comprised of three cholesteric liquid crystal compounds and a varying concentration of a soluble additive. These chemical compositions have discrete and repeatable change-of-state temperatures consistent with an empirically established formula to produce a series of change-of-state temperatures consistent with the indicated temperature points on the device. The chemicals are fully encapsulated by a clear polymeric film, which allows observation of the physical change but prevents any user contact with the chemicals. When the thermometer is placed in an environment within its measure range, such as 98.6°F (37.0°C), the chemicals in all of the cavities up to and including 98.6°F (37.0°C) change from a liquid crystal to an isotropic clear liquid state. This change of state is accompanied by an optical change that is easily viewed by a user. The green component of white light is reflected from the liquid crystal state but is transmitted through the isotropic liquid state and absorbed by the black background. As a result, those cavities containing compositions with threshold temperatures up to and including 98.6°F (37.0°C) appear black, whereas those with transition temperatures of 98.6°F (37.0°C) and higher continue to appear green” (Medical Indicators, 2006, pp. 1–2). Thus, the observed outcome is a count of cavities turned black. The measurement mechanism is an encased chemical compound that includes a varying soluble agent that changes optical properties according to changes in temperature. Amount of soluble agent can be traded off for change in human temperature to hold number of black cavities constant.

^2^The Edsphere™ technology for measuring English language reading ability employs computer generated, four-option, multiple choice cloze items built on the fly for any prose text. Counts correct on these items are converted into Lexile measures via an applicable Rasch model. Individual cloze items are one off and disposable; an item is used only once. The cloze and foil selection protocol ensures that the correct answer (cloze) and incorrect answers (foils) match the vocabulary demands of the target text. The Lexile text complexity measure and the expected spread of the cloze items are given by a proprietary text theory and associated equations. Thus, the observed outcome is a count of correct answers. The measurement mechanism is a text with a specified Lexile text complexity and an item generation protocol consistent with that text complexity measure. The text complexity measure can be traded off for a change in reading ability to hold constant the number of items answered correctly.

## References

[B1] Andrich D. (1989). Distinctions between assumptions and requirements in measurement in the social sciences, in Mathematical and Theoretical Systems: Proceedings of the 24th International Congress of Psychology of the International Union of Psychological Science, Vol. 4, eds Keats J. A. Taft R. Heath R. A. Lovibond S. H. (North-Holland: Elsevier Science Publishers), 7–16

[B2] Andrich D. (2002). Understanding resistance to the data-model relationship in Rasch's paradigm: a reflection for the next generation. J. Appl. Meas. 3, 325–359 12147916

[B3] Barrett P. (2008). The consequence of sustaining a pathology: scientific stagnation. A commentary on the target article, “Is Psychometrics a Pathological Science?” by Joel Michell. Measurement 6, 78–123

[B4] Bond T. G. Fox C. M. (2007). Applying the Rasch model. New York, NY: Routledge

[B5] Borsboom D. (2005). Measuring the Mind: Conceptual Issues in Contemporary Psychometrics. Cambridge: Cambridge University Press 10.1017/CBO9780511490026

[B6] Brennan R. L. (2011). Generalizability Theory. New York, NY: Springer

[B7] Bunge M. (2004). How does it work. Philos. Soc. Sci. 34, 182–210 10.1177/0048393103262550

[B8] Burdick D. S. Stone M. H. Stenner A. J. (2006). The combined gas law and a Rasch reading law. Rasch Meas. Trans. 20, 1059–1060

[B9] Castles A. Datta H. Gayan J. Olson R. K. (1999). Varieties of developmental reading disorder: genetic and environmental influences. J. Exp. Child Psychol. 72, 73–94 10.1006/jecp.1998.2482 9927524

[B10] Chang H. (2004). Inventing Temperature. New York, NY: Oxford University Press 10.1093/0195171276.001.0001

[B11] Cliff N. (1992). Abstract measurement theory and the revolution that never happened. Psychol. Sci. 3, 186–190 10.1111/j.1467-9280.1992.tb00024.x

[B12] Cook T. Campbell D. (1979). Quasi-Experimentation: Design and Analysis Issues for Field Settings. Boston: Houghton Mifflin

[B13] Crease R. (2004a). The greatest equations ever. Phys. World 17, 14 16381162

[B14] Crease R. (2004b). The greatest equations ever (Part 2). Phys. World 17, 19

[B15] Cronbach L. J. (1971). Test validation in educational measurement, in American Council on Education, 2nd Edn., ed Thorndike R. L. (Washington, D.C.: American Council on Education), 443–507

[B16] Elster J. (1989). Social norms and economic theory. J. Econ. Perspect. 3, 99–117 10.1257/jep.3.4.99

[B17] Fisher W. P. Jr (2010). The standard model in the history of the natural sciences, econometrics, and the social sciences. J. Phys. Conf. Ser. 238 Available online at: http://iopscience.iop.org/1742–6596/238/1/012016/pdf/1742-6596_238_1_012016.pdf

[B18] Fisher W. P. Jr. Stenner A. J. (2011). A technology roadmap for intangible assets metrology, in Paper presented in session on Fundamentals of Measurement Science, International Measurement Confederation Joint Symposium (Jena). Available online at: http://ssrn.com/abstract=1925817

[B19] Fisher W. P. Jr. Stenner A. J. (2012). Metrology for the Social, Behavioral, and Economic Sciences (Social, Behavioral, and Economic Sciences White Paper Series). Available online at: http://www.nsf.gov/sbe/sbe_2020/submission_detail.cfm?upld_id=36 (Accessed January 10, 2013).

[B20] Freedman D. A. (1997). From association to causation via regression, in Causality in Crisis? eds McKim V. R. Turner S. P. (South Bend, IN: University of Notre Dame Press), 113–161

[B21] Gay-Lussac J. L. (1802). Enquiries concerning the dilation of the gases and vapors. [Nicholson's]. J. Nat. Philos. Chem. Arts 3, 207–16, 257–267.

[B22] Grice J. W. (2011). Observation Oriented Modeling. London: Academic Press

[B23] Guttman L. (1971). Measurement as structural theory. Psychometrika 36, 329–341 10.1007/BF02291362

[B24] Hanlon S. T. Swartz C. W. Stenner A. J. Burdick H. Burdick D. S. (2012). Learning Oasis. Available onlie at: www.alearningoasis.com

[B25] Hausman D. (1998). Causal Asymetrics. Cambridge: Cambridge University Press 10.1017/CBO9780511663710

[B26] Hebra A. (2010). The Physics of Metrology: All About Instruments: from Trundle Wheels to Atomic Clocks. New York, Ny: Springer Wien

[B27] Hedström P. (2005). Dissecting the Social: On the Principles of Analytic Sociology. Cambridge: Cambridge University Press 10.1017/CBO9780511488801

[B28] Heilbron J. L. (1993). Weighing Imponderables and Other Quantitative Science Around 1800. Berkeley, California: University of California Press

[B29] Hobart J. C. Cano S. J. Zajicek J. P. Thompson A. J. (2007). Rating scales as outcome measures for clinical trials in neurology: Problems, solutions, and recommendations. Lancet Neurol. 6, 1094–1105 10.1016/S1474-442270290-9 18031706

[B30] Holland P. (1986). Statistics and causal inference. J. Am. Stat. Assoc. 81, 945–960 10.1080/01621459.1986.10478354

[B31] Irvine S. H. Kyllonen P. C. (2002). Item Generation for Test Development. Mahwah: Lawrence Erlbaum Associates

[B32] Jasso G. (1988). Principles of theoretical analysis. Sociol. Theory 6, 1–20 10.2307/201910

[B33] Kane M. T. (2006). Validation, in Educational Measurement, 4th Edn., ed Brennan R. L. (Westport: American Council on Education/Praeger), 18–64

[B34] Karabatsos G. (2001). The Rasch model additive conjoint measurement and new models of probabilistic measurement theory. J. Appl. Meas. 2, 389–423 12011506

[B35] Kintsch W. (1974). The Representation of Meaning in Memory. Hillsdale: Erlbaum

[B36] Kyngdon A. (2008). The Rasch model from the perspective of the representational theory of measurement (with commentary). Theory Psychol. 18, 89–109 10.1177/0959354307086924

[B37] Lattanzio S. Burdick D. Stenner A. J. (2012). The Ensemble Rasch Model. Durham, NC: MetaMetrics Paper Series

[B38] Lord F. M. Novick M. R. (1968). Statistical Theories of Mental Test Scores. Reading: Addison-Wesley

[B39] Luce R. D. (1998). Coalescing, event commutativity and theories of utility. J. Risk Uncertain. 16, 87–114 10.1023/A:1007762425252

[B40] Maraun M. D. (1998). Measurement as a normative practice: Implications of Wittgenstein's philosophy for measurement in psychology. Theory Psychol. 8, 435–461 10.1177/0959354398084001

[B41] McDonald R. P. (2009). Behavior domains in theory and in practice. Alberta J. Educ. Res. 49, 3

[B42] Medical Indicators (2006). Available online at: www.medicalindicators.com/pdf/NT-FC-Tech-bulletin.pdf

[B43] Messick S. (1989). Validity, in Educational Measurement, 3rd Edn., ed Linn R. L. (London: Collier Macmillan), 13–103

[B44] Michell J. (1997). Quantitative science and the definition of measurement in psychology. Br. J. Psychol. 88, 355–383 10.1111/j.2044-8295.1997.tb02641.x

[B45] Michell J. (1999). Measurement in Psychology. Cambridge: Cambridge University Press 10.1017/CBO9780511490040

[B46] Michell J. (2000). Normal science, pathological science and psychometrics. Theory Psychol. 10, 639–667 10.1177/0959354300105004

[B47] Michell J. (2005). The logic of measurement: a realist overview. Measurement 39, 285–294 10.1016/j.measurement.2005.09.004

[B48] Michell J. (2007). Bergson's and Bradley's version of the psychometricians' fallacy argument, in Paper presented at the First Joint Meeting of ESHHS and CHEIRON (University College Dublin, Ireland).

[B49] Michell J. (2012). Alfred Binet and the concept of heterogeneous orders. Front. Psychol. 3:261–273 10.3389/fpsyg.2012.00261 22912619 PMC3419461

[B50] Molenaar P. C. M. (2004). A manifesto on psychology as ideographic science: bringing the person back into scientific psychology, this time forever. Meas. Interdiscipl. Res. Perspect. 2, 201–218 10.1207/s15366359mea0204_1

[B51] Molenaar P. C. M. Newell K. M. (2010). Individual Pathways of Change. Washington, D.C.: American Psychological Association 10.1037/12140-000

[B52] Nersessian N. J. (2002). Maxwell and “the method of physical analogy”: Model-based reasoning, generic abstraction, and conceptual change, in Essays in the history and philosophy of science and mathematics, ed Malament D. (Lasalle, Illinois: Open Court), 129–166

[B53] Nersessian N. J. (2006). Model-based reasoning in distributed cognitive systems. Philos. Sci. 73, 699–709 10.1086/518771

[B54] Nersessian N. J. (2008). Creating Scientific Concepts. Cambridge, Massachusetts: MIT Press

[B55] Newton P. E. Shaw S. D. (2012). We need to talk about validity, Paper presented at the National Council on Measurement in Education, Annual Meeting (Vancouver, Canada).

[B56a] Nunnally J. C. (1978). Psychometric theory. New York, NY: McGraw-Hill

[B56] Pearl J. (1999). Reasoning with cause and effect, Paper presented at the International Joint Conference in Artificial Intelligence (Stockholm, Sweden). Available online at: https://bayes.cs.ucla.edu/IJCAI 99/ijcai-99.pdf

[B57] Pearl J. (2000). Causality: Models, Reasoning, and Inference. Cambridge, MA: Cambridge University Press

[B58] Rasch G. (1960). Probabilistic Models for Some Intelligence and Attainment Tests (Reprint, with Foreword and Afterword by B. D. Wright, Chicago: University of Chicago Press, 1980). Copenhagen, Denmark: Danmarks Paedogogiske Institut

[B59] Roche J. (1998). The Mathematics of Measurement: A Critical History. London: The Athlone Press

[B60] Schönemann P. E. (1994). Measurement: The reasonable ineffectiveness of mathematics in the social sciences, in Trends and Perspectives in Empirical Research, eds Borg I. Mohler P. (Berlin, NY: Walter de Gruyter), 149–160 10.1515/9783110887617.149

[B61] Shaw S. D. Newton P. E. (2012). Cracks in construct validity theory, Paper presented at the National Council on Measurement in Education Annual Meeting (Vancouver, Canada).

[B62] Sherry D. (2011). Thermoscopes, thermometers, and the foundations of measurement. Stud. Hist. Philos. Sci. 42, 509–524 10.1016/j.shpsa.2011.07.001

[B63] Smith R. M. (2000). Fit analysis in latent trait measurement models. J. Appl. Meas. 1, 199–218 12029178

[B64] Stenner A. J. (1994). Specific objectivity: local and general. Rasch Meas. Trans. 8, 374–375

[B65] Stenner A. J. Burdick D. S. (2011). Can psychometricians learn to think like physicists?. Meas. Interdiscipl. Res. Perspect. 9, 62–63 10.1080/15366367.2011.558797

[B66] Stenner A. J. Burdick H. Sanford E. E. Burdick D. S. (2006). How accurate are Lexile text measures. J. Appl. Meas. 7, 307–322 16807496

[B67] Stenner A. J. Smith M. III. (1982). Testing construct theories. Percept. Motor Skills 55, 415–426 10.2466/pms.1982.55.2.415

[B68] Stenner A. J. Smith M. III. Burdick D. S. (1983). Toward a theory of construct definition. J. Educ. Meas. 20, 305–316 10.1111/j.1745-3984.1983.tb00209.x

[B69] Stenner A. J. Stone M. H. Burdick D. S. (2009). The concept of a measurement mechanism. Rasch Meas. Trans. 23, 1204–1206

[B70] Stone M. H. (2002). Knox's Cube Test-Revised. Wood Dale: Stoelting

[B71] Stone M. H. Wright B. D. (1983). Measuring attending behavior and short-term memory with Knox's cube test. Educ. Psychol. Meas. 43, 803–814 10.1177/001316448304300315

[B72] Trendler G. (2009). Measurement theory, psychology and the revolution that cannot happen. Theory Psychol. 19, 579–599 10.1177/0959354309341926

[B73] Turner J. (1955). Maxwell on the method of physical analogy. Br. J. Philos. Sci. 6, 226–238 10.1093/bjps/VI.23.2262213332

[B74] von Winterfeldt D. Chung N. K. Luce R. D. Cho Y. (1997). Tests of consequence: monotonicity in decision making under uncertainty. J. Exp. Psychol. Learn. Mem. Cogn. 23, 406–426 10.1037/0278-7393.23.2.406 9080011

[B75] Woodward J. (1989). The causal/mechanical model of explanation, in Minnesota Studies in the Philosophy of Science, Vol. 13 *Scientific Explanation*, eds Salmon W. Kitcher P. (Minneapolis: University of Minnesota Press), 359–83

[B76] Woodward J. (2003). Making Things Happen. New York, NY: Oxford Press

